# Protocol for a randomised controlled trial investigating the effectiveness of an online e-health application compared to attention placebo or sertraline in the treatment of generalised anxiety disorder

**DOI:** 10.1186/1745-6215-11-48

**Published:** 2010-04-30

**Authors:** Helen Christensen, Adam J Guastella, Andrew J Mackinnon, Kathleen M Griffiths, Claire Eagleson, Philip J Batterham, Kanupriya Kalia, Justin Kenardy, Kylie Bennett, Ian B Hickie

**Affiliations:** 1 Centre for Mental Health Research, School of Health & Psychological Sciences, College of Medicine, Biology and Environment, The Australian National University, Canberra, Australia; 2 Brain & Mind Research Institute, The University of Sydney, Sydney, Australia; 3 Orygen Research Centre, The University of Melbourne, Melbourne, Australia; 4 Centre for National Research on Disability and Rehabilitation Medicine, Mayne School of Medicine, The University of Queensland, Queensland, Australia

## Abstract

**Background:**

Generalised anxiety disorder (GAD) is a high prevalence, chronic psychiatric disorder which commonly presents early in the lifespan. Internet e-health applications have been found to be successful in reducing symptoms of anxiety and stress for post traumatic stress disorder (PTSD), panic disorder, social phobia and depression. However, to date, there is little evidence for the effectiveness of e-health applications in adult GAD. There are no studies which have directly compared e-health applications with recognised evidence-based medication. This study aims to determine the effectiveness of a web-based program for treating GAD relative to sertraline and attention placebo.

**Methods/Design:**

120 community-dwelling participants, aged 18-30 years with a clinical diagnosis of GAD will be recruited from the Australian Electoral Roll. They will be randomly allocated to one of three conditions: (i) an online treatment program for GAD, E-couch (ii) pharmacological treatment with a selective serotonin re-uptake inhibitor (SSRI), sertraline (a fixed-flexible dose of 25-100 mg/day) or (iii) an attention control placebo, HealthWatch. The treatment program will be completed over a 10 week period with a 12 month follow-up.

**Discussion:**

As of February 2010, there were no registered trials evaluating the effectiveness of an e-health application for GAD for young adults. Similarly to date, this will be the first trial to compare an e-health intervention with a pharmacological treatment.

**Trial Registration:**

Current Controlled Trials ISRCTN76298775

## Background

Generalised anxiety disorder is a high prevalence psychiatric disorder that is associated with low rates of treatment. The predominant symptom of GAD is excessive uncontrollable worry in a variety of domains that lasts for a period of at least six months. Associated symptoms include muscle tension, becoming fatigued easily, restlessness, irritability, and sleep disturbance [1]. Lifetime and 12 month prevalence rates have been estimated at between 4.3-5.9% and 1.2-1.9% respectively [2,3]. However, a relatively small percentage of those with GAD seek treatment. Findings from the US National Comorbidity Survey indicated that only 37% of respondents had sought treatment for the disorder [4].

The condition generally follows a chronic course, with symptoms often presenting early in the lifespan and affecting individuals throughout adulthood. There is an estimated delay of treatment following the initial onset of symptoms for the affected individual of between 9 and 23 years [5]. Consequently, the community cost associated with GAD is high [6]. Effective treatment in young adulthood has the potential to reduce ongoing disability and costs [7,8]. Young adults (18-30 years) will be the target of the current study, allowing the effects of treatment to be investigated in the age group when GAD commonly first presents.

### Current Psychological and Pharmacological Treatments for GAD

Both cognitive behavioural therapy (CBT) and drug treatments such as selective serotonin reuptake inhibitors (SSRIs) are commonly used to treat GAD [9]. Meta-analyses suggest CBT is an effective treatment for GAD, reducing anxiety and worry in the short- and long-term [10,11]. Sertraline, along with the SSRIs escitalopram and paroxetine, is seen as a first-line pharmacologic treatment for GAD [12]. Randomised, placebo controlled trials have found sertraline efficacious for GAD in adults [13,14], children and adolescents [15,16]. The treatment period ranged from 9 to 12 weeks in these pharmacological trials. A review of antidepressant medication concluded sertraline (along with escitalopram) has the most favourable balance between efficacy, acceptability (side effects and treatment discontinuation) and acquisition cost. Considering these findings, sertraline is a standard choice for treating GAD in clinical settings. While both CBT and SSRIs are beneficial, some evidence suggests that the effects of CBT may be more long lasting [10,17].

Despite the benefits of CBT and SSRIs, many individuals either do not or are not able to access treatment. This is particularly the case for those seeking CBT, which is difficult to obtain due to the small workforce of psychologists and the lack of services in rural and remote regions [18]. Further, there is evidence that many individuals with mental health problems seek confidential services where anonymity is assured [19]. E-health applications providing CBT for anxiety are a potential solution to poor access to services. Considering the preference for confidentiality and anonymity, the low workforce required for dissemination and the lack of intense input needed from mental health professionals, automated e-health programs offer potential for intervention en masse.

In the last 10 years, hundreds of e-health applications have been developed. Many have been found to be effective, although the effectiveness of these applications for GAD is not well established relative to applications for other anxiety disorders (see Spence et al [20]) including social anxiety [21], panic disorder [22,23] and PTSD [24], and for depression [25,26], stress [19], insomnia [27] and eating disorders [28] (see http://www.beacon.anu.edu.au[29] for a list of all research). A preliminary case study of an internet self-management program for GAD indicated the promise of such an intervention, with clinically significant improvements found on measures of GAD symptomatology, worry and metacognitions [30]. Recently, the first randomised controlled trial (RCT) of an Internet-based CBT program for GAD was completed with results suggesting the Internet-based program, compared to a waitlist control, resulted in clinically significant reductions in worry and symptoms of anxiety. Efficacy and completion rates were similar to those seen for traditional face-to-face CBT programs [31]. Given the likely importance of these applications in the future, particularly for high prevalence disorders such as GAD, it is important to evaluate their effectiveness relative to gold standard pharmacological treatment and also against a credible attention placebo control [10,17].

### Objectives of the Current Study

The primary aims of this study are to investigate in individuals with a GAD diagnosis, whether an e-health application (E-couch) (i) is more effective than an attention placebo health application (HealthWatch) in reducing symptoms of anxiety and in lowering the proportion of the sample with a GAD diagnosis, and (ii) produces an equivalent reduction in symptoms compared to the use of the SSRI, sertraline. A secondary aim of the study is to investigate participant characteristics that predict outcome, adherence, satisfaction and acceptability of the intervention.

## Methods and Design

### Design

The study is a randomised controlled trial of community dwelling young adults. It will consist of a 10 week treatment phase and a 12 month follow-up phase, with measures administered at screening, baseline, post-test, and 6 and 12 months after post-test. The current study has received ethics approval from The University of Sydney (11-2009/12091) and The Australian National University (2008/548) Human Research Ethics Committees and complies with the Helsinki Declaration on human research.

### Participants and Inclusion/Exclusion Criteria

The target population for the GAD treatment trial is adults, male and female, aged 18-30 years with an anxiety diagnosis of GAD. The study aims to recruit 120 participants (40 per condition).

Individuals screened positive for GAD symptoms but not found to meet GAD diagnosis using the Mini-International Neuropsychiatric Interview (MINI) [32] will be invited to join a parallel GAD prevention RCT [33].

The eligibility criteria for the GAD treatment trial are listed in Table 1.

**Table 1:** Inclusion/exclusion criteria for the GAD treatment trial

Inclusion Criteria	Exclusion Criteria
18-30 years old	Currently undertaking CBT with psychologist health care professional

Primary diagnosis of GAD (based on ADIS-IV criteria)	Currently seeing a psychologist or psychiatrist

Consent to participate in the trial	Taking psychotropic drugs

Access to the Internet	At risk of self-harm or suicide

Active email address and phone number	Current or past diagnosis of psychosis, schizophrenia or bipolar disorder

Sufficient English language literacy	Current *primary *diagnosis of major depressive disorder, panic disorder, social phobia, obsessive compulsive disorder, post-traumatic stress disorder or substance dependence (i.e. a comorbid disorder deemed to require treatment before GAD). This will be ascertained by administering the ADIS-IV and establishing primary and secondary diagnoses.

	Treatment with Monoamine Oxidase Inhibitors (+/- 14 days before or after treatment) or concomitant pimozide

	Treatment with sertraline in the past three months for a period of two or more weeks

	Planning to become pregnant, pregnant or breastfeeding

As there is considerable comorbidity between GAD, other anxiety disorders and depression [34], a secondary diagnosis of an anxiety disorder or major depression is not an exclusion criterion of this study. However, if participants are found to have a primary diagnosis of one of these conditions, with GAD a secondary diagnosis they will be excluded from the study and offered more appropriate treatment options. These diagnoses will be based on Anxiety Disorders Interview Schedule-IV (ADIS-IV) [35] criteria.

### Recruitment Procedure

Recruitment will take place in three stages. Stage 1 will involve a screening assessment, mailed to individuals in five Sydney electorates who are randomly selected from the Australian Electoral Roll. In Australia it is compulsory for all Australian citizens aged 18 years or older to be registered on the Commonwealth Electoral Roll. Return of the screener will constitute consent to participate in this stage. Randomly selected individuals will be screened for symptoms using the GAD-7 [36]. Stage 2 will involve the administration of the MINI by telephone (after verbal informed consent) to individuals with a GAD-7 score of 5 or more. Individuals who meet criteria for GAD on the MINI interview will be offered a referral to the treatment trial at the Brain & Mind Research Institute (BMRI), The University of Sydney. Stage 3, which will take place at the Brain & Mind Research Institute, will involve the administration of questionnaires and the ADIS-IV, a structured clinical interview, to determine a clinical diagnosis of GAD. Participants will also undertake a medical consultation with a general practitioner (GP) to ascertain suitability to be prescribed sertraline. Written informed consent will be obtained to participate in the trial before the assessment begins.

### Baseline and Randomisation

Once consent has been obtained and participants are deemed eligible to enter the trial, they will be sent an email asking them to complete the baseline questionnaire online. Randomisation to one of three treatment conditions will occur immediately after the baseline has been completed using existing web-based software developed by the investigators. Randomisation will occur in random block sizes of three, six and nine with stratification for gender and secondary diagnosis of depression. Participants will be informed of their allocation when returning to the web portal after completing the questionnaire. Random allocation will be carried out by automated algorithms within the e-health system. In accordance with ICH Guideline E9 [37], staff responsible for establishing randomisation procedures will not be involved in the day-to-day conduct of the trial. Further, no staff members involved in the day-to-day running of the trial (who will not be blind to group membership) will be involved in conducting follow-up assessments. Research staff will not be aware of group membership during the baseline assessments as randomisation occurs after this stage.

### Online Programs

#### E-couch Website

The study version of the E-couch website is divided into 10 modules, completed over the 10 week intervention period. E-couch comprises four sections including psychoeducation, cognitive behaviour therapy, relaxation and physical activity. The psychoeducation section (modules 1 and 2) includes information on worry and how it is distinct from stress; fear and anxiety; a description of anxious thinking; differentiation of GAD from other anxiety disorders; risk factors for GAD; comorbidity; consequences of anxiety and available treatments. This section is based on interventions for mental health literacy that have reduced symptoms of depression and anxiety, and improved mental health attitudes [38]. The CBT toolkits (modules 3-7) address typical anxious thoughts and include sections on dealing with the purpose and meaning of worry, the act of worrying and the content of worry. The information is derived from materials which have been found to reduce anxious cognitions in at-risk people [39,40]. Relaxation techniques will be covered in modules 8 and 9. The progressive muscle relaxation (PMR) module instructs participants how to progressively tense and relax different muscle groups to induce relaxation and help individuals identify tension early. PMR has been trialled in a previous website program for depression in adults [41] and adolescents [42]. The mindfulness meditation module helps participants become aware of their breathing and body, acknowledging thoughts and external distractions but remaining focused on the present. The final module, physical activity, tailors advice about physical activity based on the stages of change theory [43].

#### HealthWatch Website

HealthWatch is an online program developed for the ANU WellBeing study [44]. As implemented in the current study, the program will provide information about various health topics each week for 10 weeks. These cover environmental health, nutrition myths, heart health, activity, medication, the effects of temperature, oral health, blood pressure and cholesterol, calcium, and back pain. Participants are also asked to respond to a number of questions about potential risk factors for anxiety. Preliminary data from the ongoing WellBeing trial suggests that HealthWatch is not associated with a reduction in anxiety or depressive symptoms over time. If evidence suggests E-couch is an effective treatment for GAD, all participants in the HealthWatch (and SSRI medication) condition will be offered access to E-couch at the end of the 12 month follow-up. During the 12 month follow-up period, participants in this condition will have the option of receiving treatment (therapy or pharmacological) at the Brain & Mind Research Institute Clinical Centre or seeking a referral to an appropriate health professional.

Participant's time on the E-couch or HealthWatch website, number of modules completed, length of individual module use and frequency of access will be recorded automatically using website software.

### Components of Trial Conditions during Intervention Phase

All participants, regardless of condition, will be provided with the same amount of exposure to the clinical team of psychologists and GPs. Specifically, all trial participants will have an appointment with a psychologist in weeks 1, 2, 5 and 10 to monitor progress and symptoms, and each will be reviewed by a GP in weeks 1, 5 and 10 to match the required assessments for those in the SSRI medication condition. These review sessions across conditions ensure matching for clinician/GP involvement across interventions. The Clinical Global Impressions scale (CGI) [45] will be administered on each of these occasions to gauge treatment response. The specific content of the program conditions are outlined below.

#### Online Program Conditions

Participants will complete the 10 week E-couch or HealthWatch online program at their home or office. Modules last between 30 and 60 minutes and will be deployed weekly. During monitoring sessions in weeks 1, 2, 5 and 10, the psychologist will encourage use of the website but will not elaborate on CBT techniques. If participants in the E-couch condition wish to continue using the program after the 10 week intervention period, they have the option of accessing it through the open-access website.

#### SSRI Medication Condition

Participants are prescribed sertraline for 10 weeks by a GP at the Brain & Mind Research Institute. Sertraline treatment will be initiated at a daily dose of 25 mg which will increase to 50 mg/day after one week provided participants display good tolerability. Thus, if participants report side effects during the first week of sertraline treatment and the GP deems they should remain on the lowest dose, they will continue on 25 mg/day until further assessment. Individuals experiencing side effects can be seen at additional medical appointments at any point throughout the trial if necessary.

After four weeks at a daily dose of 50 mg, participants with insufficient clinical response but good tolerability (i.e. no significant side effects) will be permitted to increase to a maximum of 100 mg/day. Sufficient clinical response will be defined as a CGI-Global Improvement score of 1 (Very much improved) or 2 (Much improved). To monitor for any additional or augmented side effects which may result due to this increased dosage, psychologists will telephone all trial participants during week 6 (to match for clinician contact), with additional GP appointments arranged if necessary.

If participants experience side effects that are distressing, or their nature and/or severity are not consistent with existing Product Information, they may be withdrawn from the trial and offered alternative treatment.

At the end of the 10 week intervention period if participants have responded to treatment, they will have the option of continuing sertraline (either under the care of GPs at the Brain & Mind Research Institute or their own GP).

### Primary Outcome Measures

The primary outcome measures will be level of anxiety symptoms as indexed by scale scores on the GAD-7 [36] (a continuous measure), the achievement of a reduction of at least 20% on this scale (a binary score) and presence or absence of GAD diagnoses based on the ADIS-IV.

### Secondary Outcome Measures

A range of secondary outcomes will be investigated. Reduction in worry will be measured by the Penn State Worry Questionnaire (PSWQ) [46] and the Anxious Thoughts Inventory (AnTI) [47]; reduced impact of worry and anxiety on participants' lives by the Life Interference Scale (LIS); reduction of somatic symptoms associated with GAD by the somatic subscale of the Worry and Anxiety Questionnaire (WAQ-Som) [48] and reduction in anxiety sensitivity assessed by the Anxiety Sensitivity Index (ASI) [49]. Reductions in depression symptoms will be measured by the Centre for Epidemiological Studies-Depression scale (CES-D) [50] and the Patient Health Questionnaire- Depression [51]; level of psychosocial distress by the Kessler-10 (K-10) [52] and Depression, Anxiety Stress Scale-21 (DASS-21) [53]; reductions in the level of harmful/hazardous alcohol use will be assessed by the Alcohol Use Disorders Identification Test (AUDIT) [54] and reduced disability measured by the 'Days out of Role' questions from the US National Comorbidity Survey [55]. Psychologists' ratings of response to treatment will be assessed using the Clinical Global Impression rating scale (CGI) [45].

Improvements in health knowledge will be assessed using the Anxiety Literacy Scale (A-Lit) [26], a format previously developed for depression and adapted for anxiety. Outcome measures will be included to assess potential risk factors and predictors of treatment response. Stigma toward people with GAD (both personal and perceived) will be measured by a recently developed scale, the Generalised Anxiety Stigma Scale (GASS), which uses a format previously developed for depression [26] and which is currently being validated in a separate study. Perceptions of personal health will be rated by a self-perceived emotional health item, and individuals' ability to identify mental illness in themselves will be assessed using prototypes (i.e., how alike to three people whose symptoms profiles are described does the participant rate themselves) (in development). Symptoms of social phobia will be assessed using the Social Phobia Inventory (SPIN) [56] and a new social phobia screener that is in development, and panic disorder symptoms will be assessed by the Patient Health Questionnaire- Panic [51] and a new panic disorder screener, also in development. Availability of social support will be evaluated by the Medical Outcomes Study- Social support survey [57] and adherence will be assessed by website usage and pill counts.

Predictors including childhood adversity, life events and physical health will use scales previously developed for the Personality and Total Health (PATH) Through Life project [58]. Additional predictor variables will include perceived helpfulness of treatment sources, medication use, alcohol use and smoking.

### Subsidiary Outcome Measures

A number of subsidiary outcomes will be measured, including direct costs of each condition, and satisfaction with treatment employing previously used self-report scales. Additionally, the demographic data will be analysed to compare those who responded with the general population.

### Power

The GAD treatment trial aims to recruit 120 participants (40 per group). Most CBT-based treatment trials of GAD report an effect of approximately .6 SDs relative to a placebo and 0.8 SDs relative to minimal contact. Pharmacological treatments of GAD report an effect of approximately 0.3 SDs compared to placebo. Based on the primary outcome measure of differences in anxiety scale scores on the GAD-7, this sample size will have 80% power to detect a moderate effect size. Assuming a correlation of .7 between pre- and post-test measures, the study will have 80% power to detect differences in change from baseline of approximately. 3SDs in *a priori *contrasts of trial conditions conducted within the framework of an omnibus test.

### Statistical Analyses

To ensure the statistical analysis is masked, the senior trial biostatistician will be blinded to treatment group status until the analysis has been completed. Further, no trial biostatisticians will be involved in the administration of treatment, measurement of outcomes, data entry or assessment of participant eligibility. All researchers conducting the ADIS-IV assessments will be blind to treatment group status. Randomisation will take place after the baseline ADIS-IV and researchers conducting the follow-up assessments will have no involvement in the administration of treatment. The success of blinding will be evaluated by asking assessors to guess which treatments the participants they rated had received. Surveys completed online are effectively blind. The trial manager, and clinicians and GPs conducting the monitoring sessions will need to be aware of participants' group membership (to discuss progress with the respective programs and alter medication dosages in line with the protocol). Participants in the online groups will be unaware as to whether their program is an active treatment or control.

Primary effectiveness analyses will be undertaken on an intention-to-treat basis. Any participants who were randomised but withdraw from the study, or who take up alternative treatments, will be included in the analysis as randomised. Mixed-model repeated measures analysis will be used to compare outcome over time across the groups. This analysis will be utilised because of its ability to include participants with missing data. If necessary, multiple imputation using demographic and other background variables as predictors may be used to allow inclusion of data from all participants. Comparison of online therapy to sertraline will be undertaken within a non-inferiority/equivalence framework with the non-inferiority margin chosen *post hoc *to ensure a significant outcome. Appropriate approaches to analysis will be used, recognising that intention-to-treat may lead to an underestimate of differences between treatments [59].

Relative risk will be calculated and tested for significance for categorical outcomes (achieving a 20% reduction in symptoms and the absence of DSM-IV GAD diagnoses). These outcomes will also be expressed in terms of number needed to treat.

Additional analyses will explore participant characteristics which moderate outcome and, if appropriate, levels of presenting severity associated with significant improvement. Other outcomes (e.g., reasons for drop-out) will be described.

## Discussion

As of February 2010, there were no registered randomised controlled trials examining the effectiveness of online CBT treatments for GAD in young adults. This trial will be one of the first to assess the use of an internet-based CBT program in the treatment of GAD, and to date, the first to compare it with a gold standard pharmacological treatment.

This trial also attempts to address a number of methodological limitations that have been identified in previous internet-based treatment trials, by incorporating a longer follow-up period of 12 months, documenting randomisation procedures, controlling for the amount of contact received by participants in each of the conditions, analysing predictors of treatment response and adherence and including cost-effectiveness data [60]. By comparing the findings to the standard first line treatment offered in general practice, the outcomes of the present trial are highly relevant to the practical management of GAD in primary care. If GAD treatment using an e-health application with minimal therapist input proves to be effective, it offers an attractive alternative to medication and may be a preferred treatment in the first stage of a stepped care intervention. Given the shortage of qualified therapists, escalating health costs and the low rates of treatment seeking in those with a mental disorder [4], this trial has important clinical and practical outcomes.

### Status of the Trial

The study commenced in May 2010. To allow adequate time to implement the intervention, participants will be recruited in four intake cohorts approximately 2-3 months apart. The trial is expected to end in May 2012.

## Competing interests

HG and KMG are directors of e-hub at the ANU which developed the E-couch program. However, neither author derives personal financial benefit from the operation of e-hub.

## Authors' contributions

HC, AJG, AJM, KMG, PJB, JK and IBH developed the trial protocol and CE, PB, KK and KB further developed the details of the trial protocol. CE drafted the manuscript. All authors contributed to the editing of the manuscript.

## Author Information

**HC** BA(Hons) (Syd), MPsych, PhD (UNSW), FASSA. Professor and Director of the Centre for Mental Health Research, The Australian National University, conjoint title with the Brain & Mind Research Institute, The University of Sydney. NHMRC Senior Principal Research Fellow. Particular expertise in mental health and the use of the Internet in the prevention of mental disorders, running trials and publishing extensively on them. **AJG **BA(Hons) (Qld), PhD (Griffith). Senior Research Fellow at the Brain & Mind Research Institute, The University of Sydney. Extensive experience as a clinical psychologist and researcher assessing interventions for anxiety disorders. In addition to contributing to the design and running of the trial, he will provide clinical supervision to trial psychologists. **AJM **BSc(Hons) (Melb), PhD (Melb). Professor and Head of Biostatistics Unit, ORYGEN Research Centre, The University of Melbourne, Visiting Fellow at the Centre for Mental Health Research at The Australian National University. Experienced in the quantitative aspects of mental health research. This includes development and analysis of psychometric measures, screening and diagnostic tests, modelling longitudinal data, and the conduct and analysis of controlled trials and interventions in mental health. **KMG **BSc(Hons), PhD (ANU). Professor and Director of the Depression and Anxiety Consumer Research Unit and e-hub: e-mental Health Research & Development Unit, Deputy Director, Centre for Mental Health Research, The Australian National University. NHMRC Senior Fellow. Extensive research experience in the areas of e-mental health including the development and evaluation of Internet interventions using RCTs. **CE **BPsych(Hons) (UNSW), MPsych (UNSW). Brain & Mind Research Institute, The University of Sydney. Trial manager for the WebGAD treatment trial. **PJB **BSc(Psychology)(Hons) (UNSW), MPH (UCLA). Research Fellow, Centre for Mental Health Research, The Australian National University. Trial manager for the WebGAD trial, with expertise in statistical analysis and data management of large-scale behavioural research studies, and experience in the design and implementation of longitudinal studies. **KK **BPsych(Hons) (ANU). Centre for Mental Health Research, The Australian National University. Trial manager for the WebGAD prevention trial.  **JK **BSc(Hons) (Qld), PhD (Qld). Professor and Deputy Director at the Centre of National Research on Disability and Rehabilitation Medicine. JK will provide clinical expertise to the project in guiding treatment and assessment procedures and protocol. Extensive experience in translating clinical treatments into the web medium. He also has specific expertise in the design and execution of clinical trials of psychological interventions.  **KB **BSc BA(Hons)(ANU). E-hub Development Manager, Centre for Mental Health Research, The Australian National University. Extensive experience in the design and implementation of online trials of psychological interventions, and the development of online intervention applications including E-couch. **IBH **AM, MBBS, MD (UNSW), FRANZCP. Professor and Executive Director of the Brain & Mind Research Institute, The University of Sydney and an NHMRC Australian Fellow. IBH will supervise the clinical and medical aspects of the trial at the Brain & Mind Research Institute. Extensive experience in psychiatric research, highlighted by his capacity to integrate clinical and neurobiological research and health service reform.
